# Predictive Energy-Aware Routing Solution for Industrial IoT Evaluated on a WSN Hardware Platform

**DOI:** 10.3390/s22062107

**Published:** 2022-03-09

**Authors:** Eusebiu Jecan, Catalin Pop, Ovidiu Ratiu, Emanuel Puschita

**Affiliations:** 1 Communications Department, Technical University of Cluj-Napoca, 28 Memorandumului Street, 400114 Cluj-Napoca, Romania; eusebiu.jecan@com.utcluj.ro; 2 Control Data Systems S.R.L., Liberty Technology Park, 21 Garii Street, 400267 Cluj-Napoca, Romania; catalin.pop@cds.ro (C.P.); ovidiu.ratiu@cds.ro (O.R.)

**Keywords:** predictable lifetime, energy aware, IWSN, IIoT, energy margin, ISA100.11a, routing

## Abstract

In industrial wireless sensors networks (IWSNs), the sensor lifetime predictability is critical for ensuring continuous system availability, cost efficiency and suitability for safety applications. When deployed in a real-world dynamic and centralised network, the sensor lifetime is highly dependent on the network topology, deployment configuration and application requirements. (In the absence of an energy-aware mechanism, there is no guarantee for the sensor lifetime). This research defines a conceptual model for enhancing the energy predictability and efficiency of IWSNs. A particularization of this model is the predictive energy-aware routing (PEAR) solution that assures network lifetime predictability through energy-aware routing, energy balancing and profiling. The PEAR solution considers the requirements and constraints of the industrial ISA100.11a communication standard and the VR950 IIoT Gateway hardware platform. The results demonstrate the PEAR ability to ensure predictable energy consumption for one or multiple network clusters. The PEAR solution is capable of intracluster energy balancing, reducing the overconsumption 10.4 times after 210 routing changes as well as intercluster energy balancing, increasing the cluster lifetime 2.3 times on average and up to 3.2 times, while reducing the average consumption by 23.6%. The PEAR solution validates the feasibility and effectiveness of the energy-aware conceptual indicating its suitability within IWSNs having real world applications and requirements.

## 1. Introduction

Industrial wireless sensor networks (IWSN) are deployed in harsh, hardly accessible environments to serve as control and monitoring infrastructure solutions. The sensor nodes have limited energy resources and their availability as well as maintenance cost is greatly influenced by the battery replacement procedure. Moreover, the adoption of heterogeneous nodes in terms of communication protocol, energy consumption, and initial battery level further emphasizes the concern over the network lifetime [1].

The approach towards energy-saving in IWSNs should consider the application specifics so that more efficient use of the residual energy allows network requirements to be achieved. For instance, the main characteristics of industrial IoT applications are bounded delay, deterministic communication, robustness, and security. Hence, industrial communication standards such as ISA100.11a [2] and WirelessHART [3] are poised to assure application characteristics while implementing energy efficiency techniques. Several factors are influencing the energy consumption within IWSNs, such as network topology and protocols, data acquisition, resource allocation, and radio module architecture [4]. To address them, the literature proposes the following energy-efficient mechanisms: radio optimization, data reduction, sleep/wakeup schemes, battery harvesting, and routing schemes [5,6].

In this paper, we propose the predictive energy-aware routing (PEAR) solution to enhance the network lifetime in terms of prediction, sensor node energy consumption profiles, and flexible energy balancing. The particularities of this solution closely reflect the requirements and constraints of the Industrial WSN applications. For instance, a cost-efficient battery replacement procedure should be predictable and scheduled for the entire network or cluster. To suit this, the time until the first sensor node in the network exhausts its energy is considered the network lifetime. Moreover, given the limited hardware resources in conjunction with the need for real-time hierarchical network management, a low complexity routing algorithm is required [7].

Considering industrial IoT application, the expected maximum network uptime is typically related to sensor specifications (i.e., battery capacity, consumption rate) and data update period.

The performance of the proposed routing technique is not evaluated against a theoretical maximum lifetime [8,9] but rather against the expected network or cluster lifetime thus, not targeting a consumption lower than anticipated. IIoT deployments comprise assorted sensors arguing the need for energy consumption profiles to accommodate different sensor lifetime expectancy within a WSN. The senor nodes are grouped into homogeneous consumption clusters based on the adoption of energy profiles. This approach allows for lifetime prediction and battery replacement scheduling on a per cluster basis. Furthermore, the overall network lifetime can be enhanced by balancing the energy use rate between consumption clusters. This perspective implies an increase in lifetime expectancy for the entire network at the expense of decreasing the lifetime of one or more consumption clusters.

The main contributions of this work are as follows:

- Firstly, this research gives the technical, feasibility and application details of the WSNs lifetime problematics contextualized for IIoT systems deployed in harsh industrial environments. The applicability of the related theoretical solutions is assessed indicating the need to consider the application specifics.
- Secondly, a generic conceptual model is proposed for adding energy aware solutions in IWSNs. The model presents the building blocks of energy-aware IWSNs and the method for interconnecting them in a coherent manner.
- Thirdly, the major contribution of this paper is the customization of the energy aware model targeting industrial control and monitoring, namely the PEAR solution. The proposed solution is applied for the ISA100.11a wireless industrial communication standard and is implemented on the VR950 IIoT Gateway [10] hardware platform. Enabling the PEAR solution for a 70 WSN field devices, reduces the sensors nodes overconsumption 10.4 times and extends the cluster lifetime by 21.27 and 2.5 months for low- and high-consuming WSN field devices, respectively.
- Finally, the PEAR solution provides an energy balancing mechanism between and within energy clusters. This mechanism allows the PEAR solution to reduce the average energy consumption and increase the number of field devices respecting their energy profile by 25% for a high consumption cluster.

All in all, PEAR proves to be a versatile solution, capable of adding predictable lifetime, lower overconsumption, and larger lifetime for a IWSN with multiple clusters

The structure of the paper is as follows. Section 2 contextualises the PEAR solution by presenting a comprehensive top-down analysis of the energy management solutions and energy aware routing algorithms for WSNs. Section 3 describes a conceptual model for adding energy awareness within IWSNs. Section 4 gives a detailed description of the PEAR solution in terms of model design and system integration. Section 5 presents the main performance indicators and the impact of the PEAR solution. Finally, Section 6 concludes the paper.

## 2. Related Work

One of the main concerns in the design of IWSNs is power efficiency targeting the prediction and extension of the network lifetime. While several energy efficient routing algorithms were proposed, to the best of our knowledge, no studies have been found to have a systematic approach (i.e., considering the specific requirements of an IIoT application) and a hardware evaluation platform. Moreover, the PEAR solution is assessed on a heterogeneous dual-standard WSN platform comprising the ISA100.11a and WirelessHART industrial communication protocols [11], thus further emphasizing the novelty of the study.

PEAR is designed to add energy-efficient policies suitable for industrial monitoring and control applications and respecting the ISA100.11a standard. The research carried out on routing algorithms employ simulations based on network models. Surveys such as [6,12,13] have classified the network models with respect to the traffic pattern, network structure, communication model, topology, and network management. Given this classification, the ISA100.11a network can be defined as a centralized WSN where routing and resource allocation decisions are the responsibility of a single Gateway entity. It uses a hierarchical network structure in which the sensor nodes can assume different roles namely, router and input/output. A router node is capable of forwarding the traffic packets of its neighbors while input/output nodes can route only their own data packets. A dynamic self-configuring and self-healing mesh network [14] is employed having up to five layers and respecting the convergence traffic pattern where nodes on lower layers forward the data packets of their upper-layer neighbors. Thus, the data load is cumulative and higher for nodes closer to the sink node.

Network models presented in [8,15,16] aim to monitor an area and assume that only a set of the sensing nodes need to be active at a certain time. The network is considered depleted only if the remaining sensors do not satisfy the expected monitoring performance. On the other hand, in order to reflect the demands of real IWSNs deployments we regard that all nodes should sense data during the network lifetime and that energy cannot be balanced by rotating the active set of nodes.

In what follows, PEAR will be contextualized using a top-down approach. In [1] the need for energy-efficient solutions is underlined as a part of the challenges and directions for IIoT systems. To address such demands, upper-layer designs are recognized to have a major impact. However, current research is not applicable to real IIoT environments. Among the upper-layer energy-saving mechanisms presented in [5] the routing algorithms play an important role in the efficient use of the bandwidth and battery resources [17]. Two routing paradigms are the most related to PEAR namely, cluster architectures and energy as a routing metric. A considerable amount of research has been carried on Low Energy Adaptive Clustering Hierarchy (LEACH) protocol and its variants developed in more than 15 years [18,19,20]. It is a hierarchical protocol in which a part of the sensor nodes assumes the cluster head (CH) role to coordinate the neighboring nodes forming a cluster. PEAR overcomes the drawbacks of the LEACH protocol by having a deterministic resource allocation and topology control and not employing a random load rotation among CHs. Moreover, PEAR adapts to the nodes residual energy level and is not limited to single hop communication. Thus, larger areas can be covered, and the transmission power is lower due to shorter transmission distances [21,22]. These disadvantages were addressed by LEACH-based protocols such as [23,24,25]. However, the centralized multi-hop variants imply high overheads and complexity.

Moreover, considering energy as a routing metric paradigm, a protocol such as UBERP [26] is not feasible within a real deployment due to its high algorithm complexity while the minimum transmission energy (MTE) [27,28] routing scheme gives an overall low power consumption at the price of poor network lifetime. The online maximum lifetime (OML) [29] and shortest widest path [20] techniques are better correlated with the Industrial WSN requirements by balancing the minimization of the total energy consumption and the avoidance of energy depleted nodes. Despite the use of the general terminology (i.e., maximization), these paradigms, due to the use of multimetrics, give a theoretical model for improving the network lifetime. These techniques, though more practical, are based only on simulations while PEAR takes a further step by using hardware platforms and considering real deployments requirements.

In the context of data intensive D2D (device-to-device) applications facing a spectrum shortage issue [30], the energy efficiency of an IoT system can be enhanced by 5G cellular networks. Combining short-range IoT systems such as WirelessHART and ISA100.11a with 5G capabilities provides expanded transmission range and channel bandwidth while enhancing the energy efficiency [31]. Moreover, within an industrial environment a private 5G aided IIoT systems would also address the privacy and data ownership concerns [32].

## 3. Energy Awareness and Prediction Model for IWSNs

### 3.1. Energy Prediction: Conceptual Model

**Figure 1:**
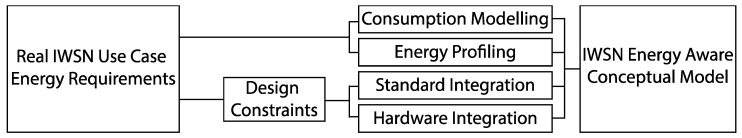
The top-down approach conceptual model of an IWSN energy-aware solution.

This paper defines a top-down approach conceptual model to provide the framework for implementing an energy awareness solution for IWSNs. The conceptual model specifies the components of an IWSN energy-aware solution and the method to interconnect them coherently as illustrated in Figure 1.

Firstly, the industrial environment and the corresponding use cases should determine the energy requirements for an IWSN. These requirements serve as input and design constraints for the consumption modelling, energy profiling, standard integration and hardware integration components. The consumption model calculates the energy expenditure associated with the wireless communication by factoring in the wireless modules characteristics such as the operation modes, energy consumption rates and the transmitted and received power. The energy profiling component models the IWSN network in terms of energy profiles and clusters. The standard integration component defines the mechanisms and methods to preserve the IWSN standardization and compatibility. To assure ensure the deployment feasibility, the hardware integration component contextualizes the energy aware solution with respect to the processing power, memory resources and coexistence with other IWSN modules.

**Figure 2:**
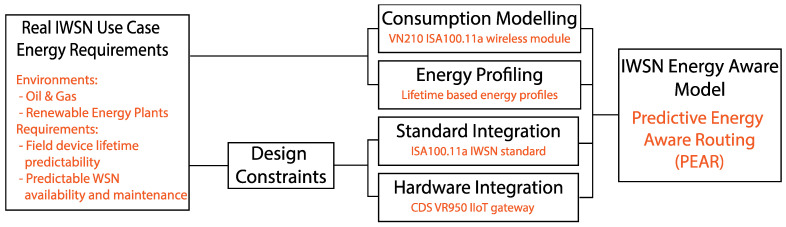
The PEAR implementation of the energy aware conceptual model respecting the ISA100.11a IWSN standard.

The PEAR solution represents a specific implementation of the energy aware conceptual model adapted for environments such as renewable energy plants and oil and gas, respecting the ISA100.11a IWSN standard and using the VR950 IIoT Gateway hardware platform. The PEAR implementation overview is given in Figure 2. 

In what follows, the PEAR implementation will be detailed in the view of the conceptual model interconnection method and components.

### 3.2. Problem Definition and Prerequisites

The PEAR solution was designed to respect the requirements, constraints and use of IWSNs deployed in real harsh and demanding environments such as oil and gas platforms and renewable energy plants. Moreover, PEAR aims to provide field device lifetime predictability and to discover the energy management potential within a standard ISA100.11a network without affecting its performance or stability. 

The PEAR design and performance criteria are based on the requirements for control and predictability of the sensor lifetime within an industrial ISA100.11a network. These requirements reflect the concerns of industrial plants for predictable availability of sensors, especially when critical or safety processes are involved and for predictable maintenance operation, such as battery replacement, to avoid high operational cost caused by frequent and unplanned maintenance procedure. The predictable lifetime and availability of IWSN sensors further prevent production discontinuity and lowers the facility downtimes.

Within an ISA100.11a network, the field device consumption and lifetime mainly depend on the battery capacity, acquisition rate and the position in the network topology. While the battery capacity is known and the acquisition rate is configurable in a dynamic mesh network, the energy consumption is dependent on the network topology. This results in a real-life case where there is a theoretical lifetime specified by the sensor manufacturer based on a standalone operation at a specific acquisition rate. However, the actual sensor lifetime cannot be controlled or predicted at sensor level when deployed within a dynamic ISA100.11a mesh network.

To accommodate such real deployment requirements, PEAR considers the following design constraints: ISA100.11a standard compatibility and IWSN hardware platform resources.

PEAR is designed to use the ISA100.11a standard attributes, objects, and mechanisms to preserve the compatibility with the ISA100.11a field devices irrespective of the manufacturer. This enhances its applicability and ease of integration with sensors from vendors such as Yokogawa [33], Honeywell [34], Draeger [35] and SpiraxSarco [36]. Moreover, PEAR uses the ISA100.11a routing and resource allocation mechanisms. To avoid a negative impact on the network performance PEAR uses the ISA100.11a standard network formation mechanism to trigger topology changes that reflect the energy constraints. This limits the PEAR possibilities to make topology changes. Therefore, PEAR does not propose a theoretical best solution approach towards energy management bur rather it provides energy awareness in the presence of routing constraints corresponding to a dynamic ISA100.11a mesh network formation.

The PEAR algorithm runs on the CDS VR950 embedded hardware platform [10] sharing processing and memory resources with other software modules, more specifically, the ISA100.11a management modules such as the system manager (SM), gateway (GW) and security manager. The topology changes triggered by PEAR consider the hardware processing limitations and do not affect the ISA100.11a network stability and performance in terms of sensor update rate.

## 4. PEAR Solution: Model Design and System Implementation

The PEAR algorithm leverages the ISA100.11a routing and resources allocation mechanisms to balance the energy consumption and to add energy awareness by signaling appropriate topology changes. The ISA100.11a standard defines a centralized network architecture where the network formation is the responsibility of the SM. The network topology and communication routes decisions are based on periodic network reports received from field devices indicating the quality of wireless links from a device perspective regarding their immediate neighbours. While the structure and interpretation of network reports are standardized, the routing and resource allocation decisions are implementation-specific.

Two routing schemes can be adopted within an ISA100.11a network, namely, source and graph routing [2]. The former specifies each hop along the path from the originating device to the destination. However deterministic, source routing is not adaptable to changes such as wireless conditions and hardware failure. Each hop along the path becomes a single point of failure affecting the system reliability and resilience to failure. Source routing is better suited for transitory communication such as the initial attachment of field devices to the ISA100.11a network. 

Graph routing specifies a communication path from one node to another along directed graphs representing a set of directed connections between devices called links. The links between two devices represent scheduled and cyclic communication opportunities during one or more timeslots (TS) of 10 ms. A neighbour is considered the device at the other end of a communication link; therefore, each device has a set of neighbour devices as candidates for packet forwarding. Routing redundancy is implemented by overlapping graphs and assigning preferred and backup neighbours indicating that the preferred link should be used even if a backup link is available sooner.

The graphs are allocated by the system manager and each field device keeps a graph table to route packets to the next applicable neighbour. For the inbound traffic, from field devices to the system manager, redundant links are allocated whereas, for the outbound traffic, from the system manager to field devices, only redundant paths (i.e., sequences of links) are allocated.

PEAR algorithm implementation adopts graph routing due to its failure resilience and configuration flexibility that makes it better suited for a dynamic ISA100.11a mesh network.

Alongside the neighbour table, each device has associated a neighbour candidates table. It specifies the quality of the physical link in terms of RSSI (received signal strength indicator, a measure of SNR) and RSQI (received signal quality indicator, a measure of bit error rate), with at least five devices in the network [2]. Each candidate can become a neighbour if it respects physical communication quality criteria, and the system manager updates the graph routing scheme to include a communication link with the candidate device.

The PEAR algorithm is constrained to generate an energy solution having available only a subset of devices, namely the ones indicated in the neighbour and candidate device tables. These tables are dynamically allocated depending on the physical environment, setup and installation. In this regard, PEAR is also a dynamic algorithm adapting to the variance in network conditions and resources.

PEAR is designed to provide energy management for an ISA100.11a network that can be abstracted as a multilayer mesh topology employing graph routing and having redundant links, preferred and backup, for all inbound traffic. Each field device is configured to have dual ISA100.11a role namely, input/output and router. Therefore, each field device can send its sensor data packets as well as forward the sensor data packets of their neighbour devices. The network topology also employs a hierarchical parent-child relationship between devices where the parent device routes the packets from its child devices, thus, increasing the traffic and energy load on the parent device. Moreover, a device can have simultaneously a parent and a child thus, the energy load is cascaded from the higher topology level to the lower ones. 

### 4.1. PEAR Energy Solution

In real use case scenarios, to ensure the economic and operational efficiency, battery- powered IWSN field devices require scheduled maintenance procedures, such as battery replacement for a subset of field devices having the same lifetime expectancy. IWSN maintenance procedures involve variable costs directly related to field device service and fixed costs associated with factory logistics and downtime. To minimize the fixed costs, the IWSN maintenance cycles should be predictable on a field device cluster basis.

To match IWSNs requirements (see section on problem definition and prerequisites), energy profiles are defined as the maximum energy consumption rate required to meet the lifetime expectation of field devices. From the PEAR algorithm perspective, the energy consumption rate is equivalated to the number of datalink protocol data units (DPDUs) transmission (Tx) and reception (Rx) opportunities per minute. Each DPDU Tx/Rx opportunity takes place in a ISA100.11a timeslot of 10 ms. A set of field devices having the same energy profile defines a profile cluster.

The PEAR algorithm aims to balance the energy consumption between and within profile clusters in order to meet the lifespan demands of all devices in a cluster. Moreover, if the energy consumption is below the Rx and Tx threshold for all devices associated with a profile, the energy consumption is not further balanced.

Depending on the communication needs and the battery capacity, the field devices have different unallocated energy margins than can be used to route data packets from other devices while respecting their expected lifetime. These margins create an energy potential in the network that is discovered and leveraged by PEAR. To simplify the scenario premises, the battery capacity is considered the same for all field devices belonging to an energy profile.

In this paper, the network lifetime is evaluated on a per energy profile basis, and it represents the time until the first field device within a profile depletes its energy capacity.

### 4.2. PEAR Consumption Model

In an ISA100.11a network, the system manager allocates cyclic communication opportunities to transmit or receive periodic sensor measurements and management data such as network reports and alarms. While the Tx and Rx rate of sensor data is based on a static configuration and is expected to be the largest source of energy consumption, the frequency and size of management packets are dynamically determined based on the network conditions and events. Moreover, due to physical phenomena such as interference and channel path attenuation, packets can be lost or received with errors resulting in additional retransmitted packets. The PEAR algorithm validates the energy threshold constraints against the cumulative Tx and Rx opportunities used, regardless of their type.

The ISA100.11a standard specifies dynamic and static energy attributes to enable energy management. The values of the EnergyLeft dynamic energy attribute is determined at network runtime and reflects the remaining battery capacity. The static energy attributes such as EnergyLife, ListenRate and TransmitRate are used by sensor manufacturers to specify the energy design of an ISA100.11a field device. The values of the static attributes are generated to emulate the manufacturer energy design specification. These attributes are the reference for the consumption thresholds and energy profile definition adopted by the PEAR algorithm.

The actual energy consumption within a time slot (TS) is dependent on the packet data size and the radio module consumption rate in different operating regimes. Since PEAR should adapt to a network that is dynamic and configurable over time, the number and size of the data packets cannot be determined beforehand. To cover the worst consumption case scenario, a publish ISA100.11a data packet with the maximum size of 115 bytes (i.e., 80 bytes for the payload and 35 bytes for the headers) is considered as the reference for consumption and lifetime estimation.

The Tx/Rx consumption rate is measured on the VN210 radio module [10] factoring in the time spent and current drawn in each operation mode namely, packet processing, clear channel assessment (CCA), Tx/Rx, operation mode switching, and acknowledgement Tx/Rx. Considering as reference a packet with the size of 115 bytes, the average consumption rate is 27.123 mA for packet transmission and 14.913 mA for packet reception. Equivalently, each Tx and Rx of a DPDU drains 7.534 nAh/149.132 nWh and 41.425 nAh/271.24 nWh respectively from the available battery capacity. Based on this consumption model, the sensor lifetime is estimated depending on the battery capacity and the number of DPDU packets per minute. For instance, a sensor node having a battery capacity of 25 Ah and maintaining the Tx and Rx consumption rate lower than 135 DPDUs/min is expected to have a three-year lifespan.

### 4.3. PEAR Algorithm Description

The PEAR algorithm is a post-network-formation mechanism examining the energy consumption across the network and signaling the appropriate changes to respect the consumption constraints of each energy profile. PEAR logically separates the network in energy clusters and uses the ISA100.11a standard management mechanisms for projecting the energy constraints into the network routing graphs.

The energy consumption can be balanced, with priority, among the field devices belonging to different profiles but also within the same profile. This approach creates higher flexibility and number of available routing changes at the possible cost of longer algorithm convergence time and a higher number of routing changes.

ISA100.11a field devices report their energy consumption using periodic communication links. If a device reports a consumption rate larger than its corresponding energy profile, the PEAR algorithm will attempt to lower the consumption by routing one of its children to another parent. The expected outcome of this change is that a device that accepts more traffic load will become the new parent of the rerouted child whereas the old parent will have fewer packets to route and thus, a lower energy consumption. The new parent candidate must have its consumption rate within its energy profile. However, due to data publish configuration or network management changes, there is no guarantee that, after accepting the traffic load from its new children, the new parent will remain within its profile consumption limits. If the new parent exceeds its profile consumption rate after a change, it becomes a candidate for energy correction.

The routing and topology algorithms are proprietary to Control Data Systems and cannot be disclosed. However, as presented in the paper, the implementation-specific configurations such as (1) energy diagnostics update period, (2) number of topology layers and (3) bandwidth allocation were not altered since PEAR aims to be an adaptable and non-intrusive algorithm.

Running an ISA100.11a network requires frequent execution cycles, under one second, for core and time-critical tasks such as data publishing as well as larger execution cycles, over one minute, for the supporting tasks such as network status reporting.

The PEAR algorithm aims to provide energy balancing support without interfering with the control and monitoring performance. The evaluation period was empirically set to one minute, which is considered large enough not to affect the efficiency and performance of the ISA100.11a management modules and small enough to provide more routing change opportunities for the PEAR algorithm. Moreover, given that CDS VR950 Gateway [10] collects new energy information from field devices every 30 s, a one minute evaluation period ensures that for each evaluation cycle the energy information is updated. Within each evaluation cycle, the energy consumption is assessed for a maximum of 10 field devices to limit the processing load.

The field devices are configured to provide periodical reports comprising the number of Tx and Rx timeslots used within an implementation-specific update period of 30 s. The reported consumption rate is compared with the corresponding energy profile. The energy consumption is considered within the profile limits if both the DPDU Tx and Rx rate are below the energy threshold.

If the energy consumption of a field device exceeds the profile threshold, PEAR initiates the energy correction procedure. When the energy profiles are properly set, the additional energy consumption is generated by routing the traffic of other devices. More specifically, the Tx timeslots used by the children devices represents the additional Rx timeslots carried by the parent device. The PEAR strategy is to unload the overconsuming device from the data traffic generated by the children having the highest Tx rate. For this, a topology change is required where the selected children reroute their packets through another parent device having the appropriate energy margin to accept additional energy load.

Firstly, the procedure creates a list with all the children of the overconsuming device arranged in descending order of the Tx rate. The Tx rate indicates the load that a field device adds to the parent. The aim is to find a new device to act as parent for a child device prioritizing the most consuming ones. For each child device, the first parent candidate is considered the backup parent. The VR950 Gateway [10] dynamically allocates backup communication links to all nodes such that the backup link can be used in the communication opportunity following a primary link failure. This preserves the network topology since these redundant communication links are allocated at network formation stage to enhance the network reliability. If the backup parent device is not suitable as the new primary parent, PEAR considers other neighbours as candidates. Among these candidates, the ones belonging to a higher consumption profile are prioritized such that, whenever possible, inter-cluster balancing is preferred over the intracluster balancing. If none of the neighbours represents a suitable new primary parent candidate PEAR repeats the new parent search for the next child device. 

**Figure 3:**
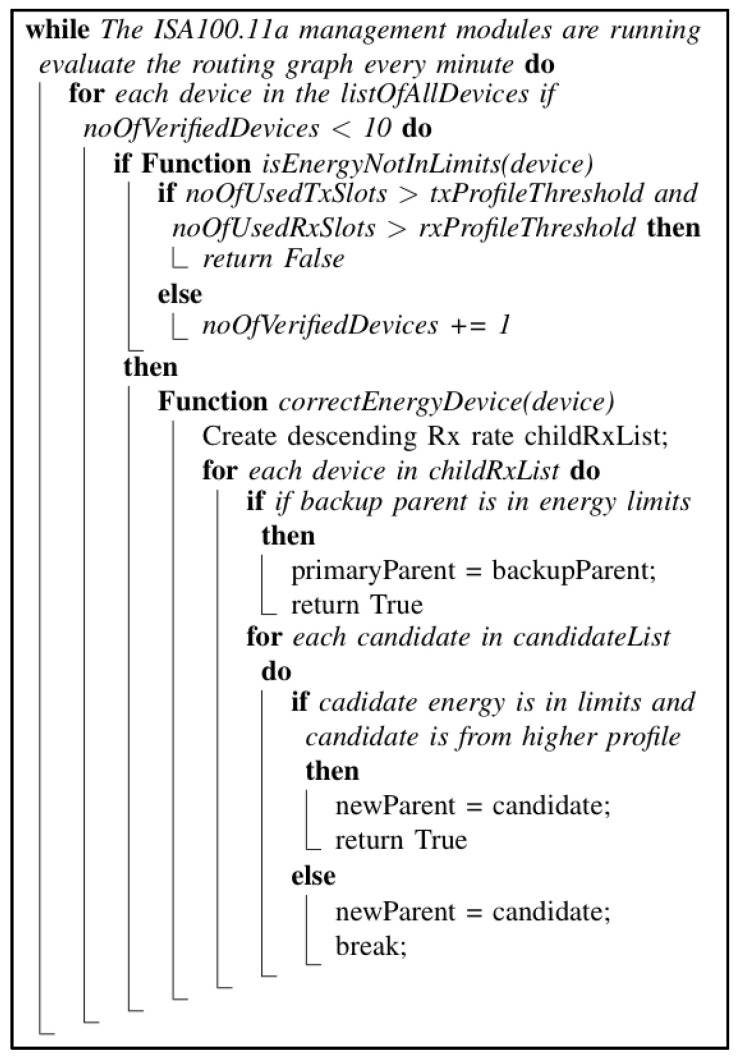
The PEAR post-network-formation pseudocode algorithm.

The PEAR post-network-formation pseudocode algorithm is described in Figure 3.

### 4.4. PEAR Impact on Network Topology Changes

As indicated in the previous section, PEAR triggers network topology changes in order to provide intercluster balancing. The network topology and its changes are dynamic and cannot be known beforehand since it is influenced by factors such as number of devices, interferences, physical position and distance between field devices. 

**Figure 4:**
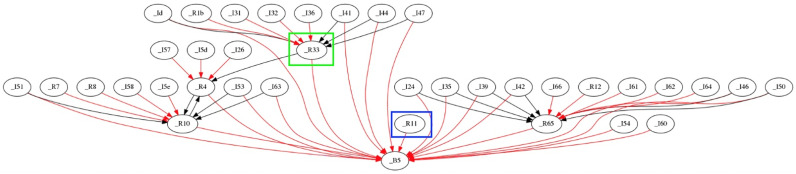
The PEAR impact on network topology formation: phase 1. The device highlighted with the green square belongs to a low energy profile whereas the device belonging to a high energy profile is highlighted with a blue square.

**Figure 5:**
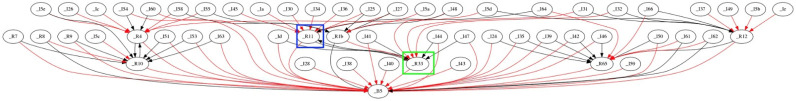
The PEAR impact on network topology evolution: phase 2. The device highlighted with the green square belongs to a low energy profile whereas the device belonging to a high energy profile is highlighted with a blue square.

**Figure 6:**
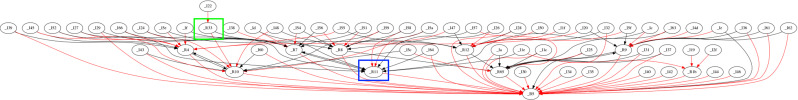
The PEAR impact on network topology evolution: phase 3. The device highlighted with the green square belongs to a low energy profile whereas the device belonging to a high energy profile is highlighted with a blue square.

To provide a representation of how PEAR influences the topology, Figure 4, Figure 5 and Figure 6 illustrate the network topology evolution in time. Phase 1 represents the inception of network formation while phase 2 and phase 3 are snapshots of its development at larger timestamps. Two examples are given: firstly, the R33 device belonging to a low energy profile (green square), and secondly, the R11 device belonging to a high energy profile (blue square). During the network formation, one can observe that R33 device had an increasing number of child devices in phase 1 and phase 2, but it was freed from the child device load. Therefore, in phase 3 the R33 device has only one child device, namely the I22 device. This emphasizes the PEAR capability to reduce the energy consumption via a topology change. On the other hand, R11 device has a direct link to the backbone router (BBR) and no child devices in phase 1. However, because R11 device can accept a higher energy load while respecting its energy profile, PEAR increases its number of child devices, thus providing intercluster balancing.

## 5. PEAR Solution: Hardware Integration and Results Evaluation

The PEAR performance will be reflected by the results of two test series employing scenarios with one, two and three energy profiles and 70 field devices. The results of the test cases where the PEAR energy balancing support is not enabled are labelled as Non-PEAR and will be used to evaluate the improvements provided by the PEAR algorithm.

### 5.1. Hardware Platform

**Figure 7:**
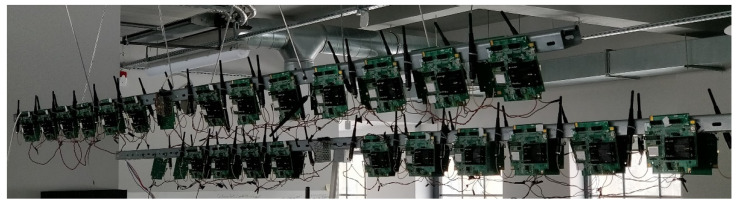
The ISA100.11a certified hardware devices employed in the experimental setup at CDS premises (Cluj-Napoca, Romania)—two out of three test batches.

The hardware platform represents the ISA100.11a infrastructure and data acquisition equipment comprising one CDS VR950 Gateway and 70 CDS VS210 field devices. Figure 7 shows the hardware used in the experiment.

The VR950 and VS210 employ the VN210 radio module as the IEEE802.15.4 based ISA100.11a 2.4 GHz wireless transceiver unit. The VR950 Gateway implements the ISA100.11a standard management modules such as the system manager, security manager and backbone router on top of an embedded Linux distribution. Moreover, the PEAR solution is integrated within the system and security management modules enabling the energy balancing support. The VR950 board is lined powered and exposes two standard ethernet ports and a serial console for backend integration and redundancy functionalities.

The VS210 is a development board implementing the ISA100.11a field device functionality including sensor data acquisition and wireless transmission. It has integrated temperature, humidity and pressure sensors whose values are acquired and transmitted every 30 s within a single data packet.

### 5.2. Scenarios Description

#### 5.2.1. Energy Profiles

The energy profiles are represented as the total number of Tx/Rx data packets (DPDU) per minute. Correspondingly, when a reference battery capacity is given, the energy profile can be represented as the expected network lifetime expressed in days, months or years. Moreover, the energy profile can be converted to mAh or mWh considering that 7.534 nAh/149.132 nWh and 41.425 nAh/271.24 nWh is required for a DPDU transmission and reception.

#### 5.2.2. Deployment Setup

To make the tests relevant, replicable and comparable, each test starts from the same initial state and follows the same deployment setup. In the initial state of each test, the gateway and all field devices are turned off. Moreover, the deployment setup is characterized by (1) the configuration of the scenario parameters and (2) the sequential powering up of the field devices on a profile cluster basis starting with the higher energy profile clusters. The field devices from the lower energy profile clusters are powered up only when 50% of field device from the higher energy cluster have joined the network. The first field devices joining the network are expected to be placed on lower topology layers and to have a higher energy consumption compared to the field devices joining the network after them. This deployment setup creates favorable inter-cluster energy balancing conditions, thus eliminating the deployment bias in energy balancing and relevantly emphasizing the PEAR performance and impact.

#### 5.2.3. Terms and Key Performance Indicators

The following key performance indicators were defined to evaluate the performance impact of the PEAR algorithm.

Overconsumption: The PEAR performance in terms of energy balancing will be characterized by the overconsumption metric. It represents the average amount of energy consumption of a field device exceeding the energy profile constraints. The cluster overconsumption represents the total energy consumption exceeding the energy profile considering all devices within a cluster. The overconsumption metric is expressed in mWh and indicates, on a field devices or cluster basis, the efficiency of the energy resources usage in terms of energy balancing, predictability, and cluster lifetime. 

Sink nodes: The sink nodes are the field devices placed on lower topology layers and routing data packets from multiple child devices.

Energy margin: The analysis of a network with one cluster reveals that the average energy consumption is 66.12 mWh/day with a standard deviation of 5.26 mWh/day corresponding to a coefficient of variation of 7.41%, regardless of the energy profiles being set or the number of topology changes. When describing the PEAR performance, the term energy margin will be used to indicate the difference between the energy profile and the average field device consumption. The energy margin value will not be used to indicate the PEAR performance but rather as a tool for interpreting the results.

Success rate: The success rate represents the percentage of the field devices within a cluster respecting the energy profile.

PEAR stability: The PEAR stability state is achieved when all possible energy balancing topology changes have been exhausted. On the other hand, the PEAR instability state is characterized by a cyclic loop of topology changes that do not result in lower overconsumption jumps or an overall better energy balancing.

### 5.3. The One Profile Test Series

The first scenario employs 70 field devices, one profile cluster and multiple energy profiles. The battery capacity is considered 25 A equivalated to 90 Wh of stored energy. Considering all one profile cluster tests, the average number of Tx and Rx packets is 110 and 134 DPDUs/min, respectively which represents 49.8 μWh/min. The average consumption would correspond to an energy profile of 118 DPDUs/min.

The performance and impact of the PEAR algorithm are given in comparison with a similar test where PEAR is not enabled labeled as Non-PEAR. Moreover, the PEAR performance evolution will be analyzed at intermediary stages marked by the number of topology changes. The test scenario aims to (1) demonstrate the PEAR capability to add energy awareness within a profile cluster and (2) to give an empirical baseline of the overall network energy needs to assure the WSN performance requirements. The network energy baseline will be referenced when defining the test scenarios with two and three profile clusters. 

The Table 1 below shows the PEAR success rate as the total field devices respecting the energy constraints for a series of energy profiles. More specifically, it represents the rate of the field devices having the average number of Tx/Rx packets per minute lower than the corresponding energy profile. This metric is evaluated for multiple energy profiles ranging from 100 DPDUs/min to 450 DPDUs/min. When all 70 field devices have the energy profile set to 450 DPDUs/min, PEAR achieves a 100% success rate. However, when exceeding the 200 DPDUs/min profile, an increase of the energy profile has a low impact on the number of devices respecting the energy constraints. A 250 DPDUs/min increase in the energy profile determines only a 10% improvement of the success rate. This behavior appears when PEAR cannot lower the energy consumption of a sink field device because it does not have a better topology alternative without causing network instability. On the other hand, for profiles lower than 200 DPDU/min, a small increase in the energy profile generates a high increase in the success rate. This demonstrates that PEAR can rapidly and efficiently balance the consumption when the energy margin is positive.

**Figure 8:**
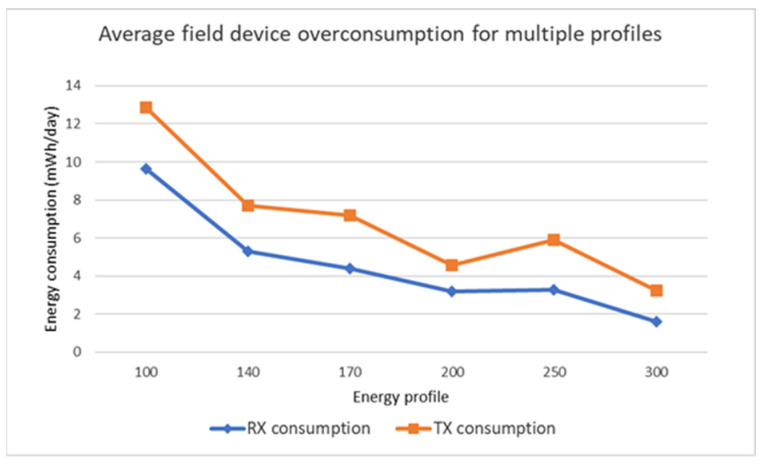
Average field device energy consumption per day exceeding the energy profile.

The Figure 8 depicts the average daily energy overconsumption of a field device considering different energy profiles. It can be observed that PEAR can significantly improve energy prediction and balancing when the energy profile is set above the average consumption of 110 Tx and 134 Rx DPDUs/min. An increase in the energy profile from 100 to 200 DPDUs/min results in a three times overconsumption decrease whereas an increase of the energy profile from 200 to 300 DPDUs/min results in less than two times overconsumption decrease. The nonlinear overconsumption improvement is determined by the PEAR ability to balance the energy within the profile cluster. In the case of an energy profile set to 250 DPDUs/min the overconsumption of the Tx communications increases when compared with the 200 DPDUs/min profile setting. This result reflects a dynamic network resources allocation mechanism where a few devices have a large energy consumption that PEAR cannot adjust without affecting the network stability and performance.

**Figure 9:**
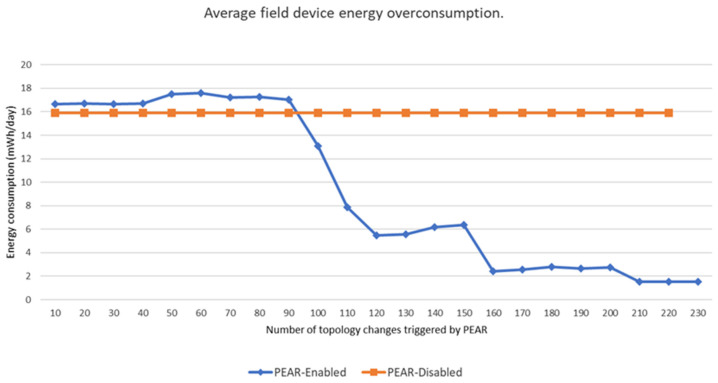
Average field device overconsumption for a 24-month profile (i.e., 200 DPDU/min) with respect to the number of topology changes triggered by PEAR.

A more comprehensive view of the PEAR workings and performance is given in Figure 9 by evaluating the average field device overconsumption for one profile cluster with 70 devices and a 200 DPDUs/min (i.e., 24 months lifetime) energy profile. The energy overconsumption is given at several intermediary stages concerning the number of topology changes triggered by PEAR. Moreover, the performance is assessed in comparison with a similar test where the PEAR support is not enabled. 

The PEAR impact on energy balance and overconsumption takes place in jumps. More specifically, three jumps can be observed namely, at 120, 160 and 210 topology changes. Each jump is preceded by an increase in energy overconsumption. This is the expected behavior since PEAR accepts intermediary energy inefficiencies for the benefit of more energy balanced network resources allocation. When trying to lower the energy consumption of a field device, the PEAR choices are limited by the set neighbours of each device. This leads to cases where an overconsuming field device cannot be freed from its child nodes due to limited choices. Therefore, PEAR triggers intermediary network topologies that create new opportunities to free the overconsuming field devices. With these opportunities PEAR can efficiently balance the energy consumption as it is illustrated by the overconsumption jumps. 

In terms of performance, PEAR reduces the overconsumption 10.4 times while having the average field device energy consumption only 1.8% larger compared with the Non-PEAR variant. This observation emphasizes that PEAR does not aim to reduce the overall energy consumption but rather to enable predictable energy consumption by balancing the energy consumption across the network. This observation emphasizes that PEAR prioritizes the predictability of energy consumption and the energy balancing across the network.

The Table 2 comprises the results of six tests showing the number of topology changes and the time to stability for a 70 devices profile cluster having different energy profiles. It shows that for negative and small energy margins (e.g., under 50 DPDUs/min) PEAR reaches instability states whereas for larger energy margins PEAR reaches a stable state in a shorter time and with a smaller number of topology changes. Moreover, for an energy margin larger than 130 DPDUs/min the impact on the number of topology changes and time to stability decreases. This nonlinear behavior emphasizes on the PEAR ability to efficiently trigger the topology changes and have the most impact within a 130 DPDUs/min energy margin.

### 5.4. The Multiple Profiles Test Series

In what follows, the PEAR performance is evaluated based on two test scenarios employing two and three profile clusters, respectively. The PEAR impact in terms of energy balancing and predictability will be evaluated by comparing the results of similar tests with and without the energy balancing support.

The scenario splits the 70 field devices in two profile clusters having the same publishing and network configuration. The clusters are differentiated based on the expected lifetime and number of field devices per cluster. The high-consuming cluster has the energy profile set to 400 DPDUs/min (i.e., one year lifetime) and the low consuming cluster has the energy profile set to 135 DPDUs/min (i.e., 3 years). One can observe from Table 2 that the three-year profile cluster having the energy margin of 20 DPDUs/min, does not reach stability and has a cluster overconsumption of 15.182 mWh/day. The two energy profiles reflect a corner case where the life expectancy difference between clusters is two years, and the low cluster could not respect the energy constraints using only within-cluster energy balancing.

The first scenario further employs seven cluster configurations concerning the distribution of the field devices per each profile cluster to give a more comprehensive view on the PEAR impact. The field device distribution varies from fifty high-profile devices and twenty low-profile devices labeled as H50L20 to only five devices in the high in profile and 65 in the low-profile labeled as H05L65. 

The configurations are detailed in the Table 3.

**Figure 10:**
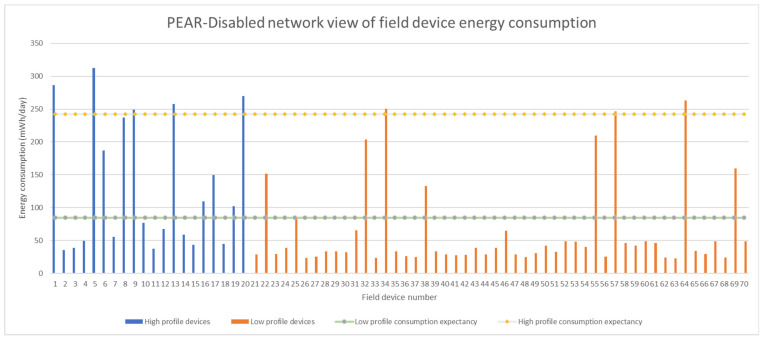
Average field device energy consumption for all devices in the network differentiated by the energy profile when PEAR is enabled.

**Figure 11:**
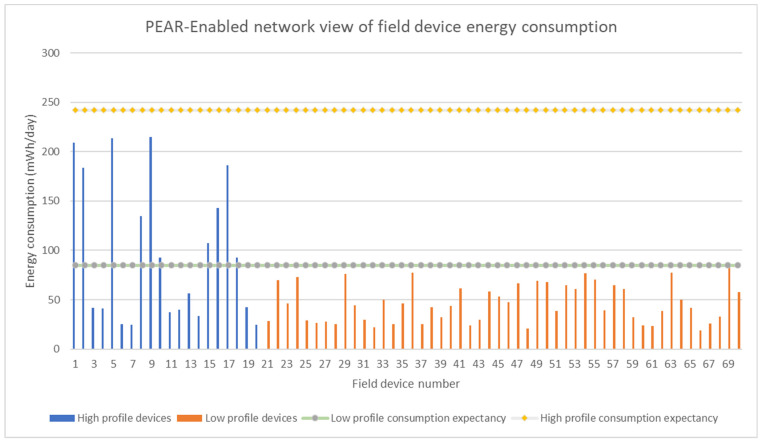
Average field device energy consumption for all devices in the network differentiated by the energy profile when PEAR is disabled.

The Figure 10 and Figure 11 represents the average field device consumption expressed in mWh/day for the PEAR and Non-PEAR variants of the H20L50 test. Moreover, the figures show the field devices distribution per cluster and their corresponding energy profile. The PEAR algorithm aims to provide predictable energy consumption by limiting the average energy consumption of each field device to respect the 12 months and 36 months cluster lifetime expectancy. The cluster lifetime is determined by the field device with the highest energy consumption therefore the overconsuming devices determine the noncompliance of the entire cluster concerning lifetime. For the H20L50 test, PEAR stability state was reached in 346 min after triggering 47 network topology changes. Therefore, the three-year lifetime expectancy that could not be achieved on a single cluster basis was respected through PEAR intercluster energy balancing.

The Figure 11 gives an overview of the PEAR impact on field device lifetime predictability. All field devices respect their energy profile while not aiming to further balance the energy after lifetime expectancy has been achieved. 

On the other hand, when the energy balancing support is not provided, as shown in Figure 10, one can observe that 18.5% (i.e., 13 devices) of the field devices do not respect the energy profile. Furthermore, three devices from the low cluster exceed not only their energy profile but also the high energy profile. This confirms that an energy balancing favorable deployment does not result in an intra- or intercluster energy balancing.

The comparison between the PEAR and Non-PEAR variants of the H20L50 test is given on a cluster basis and considers metrics such as lifetime, average energy consumption and success, as illustrated in Table 4. The Non-PEAR profile clusters lifetime is 11.23 and 9.46 months representing 25.46 and 2.5 months less than the expected cluster lifetime, respectively. Contrastingly, the PEAR clusters lifetime is greater than the corresponding profile lifetime with 5.7 and 53.5 days, respectively. Furthermore, the PEAR low cluster lifetime is 3.2 times larger compared with the Non-PEAR variant. This demonstrates the PEAR ability to balance the energy consumption within the network in order to provide cluster lifetime predictability.

The PEAR algorithm also reduces the average energy consumption with 23.6% for the lower cluster and with 25% for the higher cluster. This indicates that when multiple profiles are defined, PEAR further enhances the use of the network energy resources by lowering the overall energy consumption. 

The success rate metric shows that without the energy balancing support provided by PEAR only 84% of the low-profile devices and 75% of high-profile devices respect their intended life expectancy. Thus, the overconsuming field devices represent a significant percentage of the total field devices arguing that PEAR energy balancing is critical for assuring predictable field device lifetime within a dynamic large mesh network.

**Figure 12:**
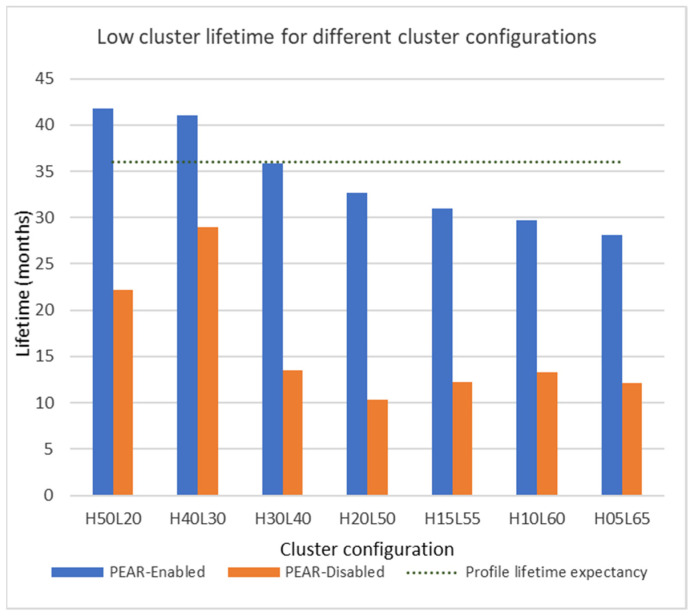
Comparison between PEAR-Enabled and PEAR-Disabled low cluster lifetime for different cluster configurations.

The low cluster lifetime for all two-profile test configurations is given in Figure 12 including the H20L50 configuration that was detailed previously. For each test configuration, the lifetime of the PEAR and Non-PEAR variants is depicted together with an indication of the low-profile expected lifetime. 

The distribution of the field devices per profile clusters gives a measure of the inter-cluster energy balancing opportunities in terms of energy margin and topology choices. More specifically, larger high-consumption clusters give better opportunities for the PEAR algorithm due to a higher overall accepted energy consumption and a larger number of field devices that can reroute data packets without exceeding their energy profile. The PEAR low-profile lifetime is larger as the high-profile cluster is larger whereas the Non-PEAR results are not correlated with the high cluster size. These results indicate that PEAR dynamically capitalizes on different network resources and configurations to improve the cluster lifetime and predictability.

The test configuration considers corner cases where the high cluster size is as large as 50 devices and as small as five devices. However, regardless of the configuration, the PEAR algorithm determines an average of 2.31 times larger cluster lifetime compared with the Non-PEAR variant. This observation also emphasizes that PEAR can provide energy balancing (1) within large clusters of 65 devices, (2) between clusters with 24 months configured lifetime difference, and (3) for different field device distributions per cluster.

The energy profile is respected for the PEAR test configurations where the low-profile field devices represent up to 71.5% of the total number of devices. A larger low-cluster size creates simultaneously two energy balancing constraints namely, (1) a greater need for intercluster energy balancing and (2) a smaller number of devices from the higher profile that can act as sink nodes. Moreover, as indicated in “Scenario 1”, an energy profile of 150 DPDUs/min does not give an energy margin large enough for a 70 devices cluster to have a 32 months lifetime. Consequently, PEAR enables a small number of high-profile nodes to enhance the lifetime of large low-profile cluster.

Table 5 gives the cluster overconsumption for all test configurations. The PEAR energy balancing support eliminates or lowers the cluster overconsumption by at least one order of magnitude thus, confirming the PEAR ability to discover and exploit the energy balancing potential within a network. 

A configuration having a smaller number of high-profile devices has a lower average energy margin per network and a lower intercluster balancing potential. Therefore, the need for using the energy potential in the network is greater when distributing fewer devices in the high cluster. The results in the Table 5 confirm this by emphasizing that the Non-PEAR low cluster overconsumption is 80.19 mWh/day for a H50L20 configuration and 1334.50 mWh/day for a H05L65 configuration. Moreover, PEAR discovers and exploits more the energy balancing potential as the need is higher and the average energy margin decreases. For instance, PEAR lowers the overconsumption with only 14.35 mWh/day for the H40L30 configuration. However, for the H05L65 configuration when the average energy margin is the lowest it reduces the cluster overconsumption almost with 1305.49 mWh/day, which is 90 times more compared with H40L30 configuration.

The second scenario comprises 70 field devices distributed in three profile clusters. The clusters are differentiated by the number of field devices and the energy profiles of 12, 24 and 48 months, respectively. The reference battery capacity is considered 20 Ah or, equivalently 72 Wh. The scenario parameters are summarized in Table 6.

The Table 7 shows the lifetime, average consumption, overconsumption and the success rate for each profile cluster. Moreover, the PEAR and Non-PEAR test variants are considered in order to facilitate performance evaluation. 

The low cluster devices are the last ones to join the network thus, being placed on higher topology layers and having less routing load. Due to the favorable deployment setup, no further topology changes are required for the low-profile devices to respect their intended lifetime. However, the deployment setup alone, while advantageous, does not assure compliance with the energy requirements for higher power consuming clusters. The PEAR algorithm improves the mid- and high cluster lifetime with 79% and 23%. The Non-PEAR results show that 25% of the mid-cluster devices do not respect the energy profile with an average overconsumption per field device of 52.6 mWh/day compared with the 97.5% success rate and average overconsumption of 7.3 mWh/day for the PEAR variant. The 7.2 times less average overconsumption argues the PEAR capacity to balance the energy not only between clusters but also within a cluster profile even for the overconsuming devices.

## 6. Conclusions

One of the main concerns for IWSNs is related to the efficient use of energy and the lifetime predictability of the sensors deployed in the field. IWSN energy efficiency solutions should be tailored to the specifics of the application and should not impact the performance of industrial IoT systems. For instance, the ISA100.11a and WirelessHART industrial communication standards are designed to ensure deterministic data collection, bounded delay, self-healing communication paths and data security. They serve as industrial control and monitoring systems carrying time and integrity critical data. 

This research defines a top-down approach framework for adding energy awareness within IWSNs. In particular, predictive energy-aware routing (PEAR) implements this framework for IWSNs deployed in critical and harsh environments, respecting the ISA100.11a communication standard and embedded on the VR950 Gateway platform.

PEAR’s scope is to provide predictable sensor lifetime and observe the field device energy profile by the means of energy-aware routing.

The impact and performance of PEAR are evaluated on an ISA100.11a network comprising 70 field devices logically split into one, two or three profile clusters. Moreover, key performance indicators such as cluster lifetime, overconsumption, average consumption, and success rate are compared for PEAR and Non-PEAR variants, respectively. 

The results show that PEAR adjusts the network topology and routing paths to ensure the expected cluster lifetime for network configurations with one or multiple clusters. Furthermore, multiple field device distributions were considered, further emphasizing the PEAR solution relevance for a variety of deployment configurations. 

Concluding, if the energy margin (defined as the difference between the energy profile and the average field device consumption) is at least 50 data packets/minute, the PEAR solution can ensure the expected cluster lifetime. Moreover, PEAR extends the cluster lifetime by up to 3.2 times while reducing the average energy consumption by 23.6% and the total overconsumption 10.4 times after triggering 210 network topology changes.

In future work, the energy awareness conceptual model could be particularized for other WSN applications by using a top-down approach. Finally, PEAR could be extended to implement additional IWSNs standards such as WirelessHART aiming for a dual-standard PEAR solution.

## Figures and Tables

**Table 1:** The PEAR success rate with respect to the energy profile.

Energy Profile (DPDUs/min)	Success Rate (%)	Number of Overconsuming Devices
100	48.57	36
135	77.14	16
170	84.28	11
200	90	7
250	92.85	5
300	95.71	3
400	97.14	2
450	100	0

**Table 2:** The number of topology changes and the time to stability.

Energy Profile [DPDUs/min]	Energy Profile [Months]	Energy Margin [DPDUs/min]	Number of Topology Changes	Time to PEAR Stability [min]
100	48	−20	>400	No stability
135	36	20	>400	No stability
170	30	50	240	708
200	24	80	231	643
250	19	130	32	87
300	16	180	19	71
400	12	280	7	31

**Table 3:** The distribution of the field devices per each profile cluster.

Configuration	High Cluster Size (1-Year Lifetime Cluster)	Low Cluster Size (3 Years Lifetime Cluster)	High Cluster Size Distribution [%]	Low Cluster Size Distribution [%]
H50L20	50	20	71.43%	28.57%
H40L30	40	30	57.14%	42.86%
H30L40	30	40	42.86%	57.14%
H20L50	20	50	28.57%	71.43%
H15L55	15	55	21.43%	78.57%
H10L60	10	60	14.29%	85.71%
H05L65	05	65	7.14%	92.86%

**Table 4:** The comparison between the PEAR and Non-PEAR per profile cluster for the H20L50 distribution.

Profile Cluster	Energy Balancing	Expected Lifetime [Months]	Lifetime [Months]	Average Energy Consumption [mWh/day]	Success Rate (%)
Low	PEAR	36	36.69	32.6	100%
	Non-PEAR		11.23	11.23	84%
High	PEAR	12	13.76	1.37	100%
	Non-PEAR		9.46	1.83	75%

**Table 5:** The PEAR and Non-PEAR cluster overconsumption per test configuration.

Test Configuration	Low Cluster Overconsumption [mWh/day]		High Cluster Overconsumption [mWh/day]	
	PEAR	Non-PEAR	PEAR	Non-PEAR
H50L20	0	80.19	0	45.20
H40L30	0	14.35	0	147.34
H30L40	0	569.93	0	20.19
H20L50	0	915.88	0	173.37
H15L55	11.29	968.97	0	14.30
H10L60	54.28	648.30	0	0
H05L65	29.01	1334.50	0	0

**Table 6:** The three profile cluster scenario parameters.

Profile Cluster	Number of Devices	Energy Profile [months]	PEAR Profile Threshold [DPDUs/min]
Low cluster	20	48	80
Mid cluster	40	24	160
High cluster	10	12	325

**Table 7:** The comparison between the PEAR and Non-PEAR for the three profile cluster scenario.

Profile Cluster	Energy Balancing	Lifetime (Months)	Average Energy Consumption [mWh/day]	Cluster Overconsumption [mWh/day]	Success Rate (%)
Low	PEAR	54.2	23.25	0	100%
	Non-PEAR	54.2	24.68	0	100%
Mid	PEAR	22.2	34.97	7.30	97.5%
	Non-PEAR	12.4	49.07	526.03	75%
High	PEAR	12.1	91.34	0	100%
	Non-PEAR	9.8	94.92	136.72	70%

## Data Availability

Not applicable.

## References

[1] Sisinni E., Saifullah A., Han S., Jennehag U., Gidlund M. (2018). Industrial internet of things: Challenges, opportunities, and directions. IEEE Trans. Ind. Inform..

[2] (2011). Wireless Systems for Industry Automation—Process Control and Related Applications.

[3] (2016). Industrial Communication Networks—Wireless Communication Network and Communication Profiles—WirelessHART.

[4] Chen Y., Zhao Q. (2005). On the lifetime of wireless sensor networks. IEEE Commun. Lett..

[5] Rault T., Bouabdallah A., Challal Y. (2014). Energy efficiency in wireless sensor networks: A top-down survey. Comput. Netw..

[6] Pantazis N.A., Nikolidakis S.A., Vergados D.D. (2013). Energy-efficient routing protocols in wireless sensor networks: A survey. IEEE Commun. Surv. Tutor..

[7] Du R., Gkatzikis L., Fischione C., Xiao M. (2018). On maximizing sensor network lifetime by energy balancing. IEEE Trans. Control. Netw. Syst..

[8] El-Fouly F.H., Ramadan R.A. (2020). Real-Time Energy-Efficient Reliable Traffic Aware Routing for Industrial Wireless Sensor Networks. IEEE Access.

[9] Chang J.H., Tassiulas L. (2004). Maximum lifetime routing in wireless sensor networks. IEEE/ACM Trans. Netw..

[10] CDS VersaRouter 950—Control Data Systems. https://www.cds.ro.

[11] Jecan E., Pop C., Padrah Z., Ratiu O., Puschita E. A dual-standard solution for industrial Wireless Sensor Network deployment: Experimental testbed and performance evaluation. Proceedings of the 2018 14th IEEE International Workshop on Factory Communication Systems-Proceedings (WFCS).

[12] Anwar A., Sridharan D. (2015). A Survey on Routing Protocols for Wireless Sensor Networks in Various Environments. Int. J. Comput. Appl..

[13] Al-Karaki J.N., Kamal A.E. (2004). Routing techniques in wireless sensor networks: A survey. IEEE Wirel. Commun..

[14] Zurawski R. (2014). Industrial Communication Technology Handbook.

[15] Akkaya K., Younis M. An energy-aware QoS routing protocol for wireless sensor networks. Proceedings of the 23rd International Conference on Distributed Computing Systems Workshops (ICDCSW).

[16] Misra R., Mandal C. (2009). Rotation of CDS via connected domatic partition in ad hoc sensor networks. IEEE Trans. Mob. Comput..

[17] Singh S.K., Kumar P., Singh J.P. (2017). A Survey on Successors of LEACH Protocol. IEEE Access.

[18] Boyinbode O., Le H., Mbogho A., Takizawa M., Poliah R. A survey on clustering algorithms for wireless sensor networks. Proceedings of the 13th International Conference on Network-Based Information Systems (NBiS).

[19] Zhou H.Y., Luo D.Y., Gao Y., Zuo D.C. (2011). Modeling of Node Energy Consumption for Wireless Sensor Networks. Wirel. Sens. Netw..

[20] Alghamdi T.A. (2020). Energy efficient protocol in wireless sensor network: Optimized cluster head selection model. Telecommun. Syst..

[21] Heinzelman W., Chandrakasan A., Balakrishnan H. Energy-efficient communication protocol for wireless microsensor networks. Proceedings of the 33rd Annual Hawaii International Conference on System Sciences.

[22] Zhang H., Zhang S., Bu W. (2014). A Clustering Routing Protocol for Energy Balance of Wireless Sensor Network based on Simulated Annealing and Genetic Algorithm. Int. J. Hybrid Inf. Technol..

[23] Saminathan A.G., Karthik S. (2013). DAO-LEACH: An Approach for Energy Efficient Routing based on Data Aggregation and Optimal Clustering in WSN. Life Sci. J..

[24] Chen J., Shen H. MELEACH-L: More energy-efficient LEACH for large-scale WSNs. Proceedings of the 2008 International Conference on Wireless Communications, Networking and Mobile Computing (WiCOM).

[25] Zytoune O., El Aroussi M., Aboutajdine D. (2010). A Uniform Balancing Energy Routing Protocol for Wireless Sensor Networks. Wirel. Pers. Commun..

[26] Ettus M. System capacity, latency, and power consumption in multihop-routed SS-CDMA wireless networks. Proceedings of the RAW-CON 98, 1998 IEEE Radio and Wireless Conference (Cat. No.98EX194).

[27] Toh C.K. (2001). Maximum battery life routing to support ubiquitous mobile computing in wireless ad hoc networks. IEEE Commun. Mag..

[28] Park J., Sahni S. (2006). An online heuristic for maximum lifetime routing in wireless sensor networks. IEEE Trans. Comput..

[29] Mohanoor A.B., Radhakrishnan S., Sarangan V. (2009). Online energy aware routing in wireless networks. Ad Hoc Netw..

[30] Humayun M., Jhanjhi N.Z., Alruwaili M., Amalathas S.S., Balasub- Ramanian V., Selvaraj B. (2020). Privacy protection and energy optimization for 5G-aided industrial internet of things. IEEE Access.

[31] Mao W., Zhao Z., Chang Z., Min G., Gao W. (2021). Energy-Efficient Industrial Internet of Things: Overview and Open Issues. IEEE Trans. Ind. Inform..

[32] Aijaz A. (2020). Private 5G: The Future of Industrial Wireless. IEEE Ind. Electron. Mag..

[33] Yokogawa Electric Corporation Yokogawa Corporation Website. https://www.yokogawa.com.

[34] Honeywell International Inc Honeywell International Website. https://www.honeywell.com/us/en.

[35] Drägerwerk AG Drägerwerk Website. https://www.draeger.com.

[36] Spirax-Sarco Engineering plc Spirax-Sarco Website. https://www.spiraxsarcoengineering.com.

